# The Amyloidosis Forum: a public private partnership to advance drug development in AL amyloidosis

**DOI:** 10.1186/s13023-020-01525-2

**Published:** 2020-09-29

**Authors:** The Inaugural Amyloidosis Forum Panelists, Melanie Blank, Michelle Campbell, John O. Clarke, Raymond Comenzo, Laura M. Dember, Angela Dispenzieri, Sharmila Dorbala, Preston Dunnmon, Douglas V. Faller, Rodney H. Falk, Nicole Gormley, Kristen Hsu, Carol D. Karp, Heather Landau, Jessica L. Lee, Isabelle Lousada, Michelle L. Mauermann, Mathew Maurer, Vaishali Sanchorawala, James Signorovitch, Kimberly Smith, Ashutosh D. Wechalekar, Brendan M. Weiss, Michelle K. White, Isabelle Lousada

**Affiliations:** Amyloidosis Research Consortium, 320 Nevada Street, Suite 210, Newton, MA 02460 USA

**Keywords:** AL amyloidosis, Light-chain amyloidosis, Primary amyloidosis, Drug development

## Abstract

**Background:**

Immunoglobulin light chain (AL) amyloidosis is a rare, multi-systemic disorder characterized by two disease processes: an underlying plasma cell dyscrasia that provides the source of pathologic light chains, and the resulting organ dysfunction caused by deposition of amyloid light chain fibrils. There are no FDA approved treatments for AL amyloidosis; regimens developed for multiple myeloma are used off-label to treat the plasma cell disorder and no therapies are directed at organ deposition. Thus, an unmet medical need persists despite advances in disease management. A public-private partnership was recently formed between the Amyloidosis Research Consortium (ARC) and the US Food and Drug Administration (FDA) to bridge scientific gaps in drug development for the treatment of AL amyloidosis.

**Main Body:**

The inaugural Amyloidosis Forum was convened at FDA on 12 November 2019 and led by a multidisciplinary panel of physicians, health outcomes professionals, and representatives from the FDA, ARC, and pharmaceutical companies. Patients provided important perspectives on the pathway to diagnosis, challenges of rigorous treatment, and the burden of disease. The panel reviewed the epidemiology, pathobiology, and clinical features of AL amyloidosis. Hematologic characteristics, staging systems, and response criteria were examined with clear consensus that a “deep response” to plasma cell-directed treatments was critical to overall survival. Emphasis was placed on the heterogeneous clinical phenotypes of AL amyloidosis, including cardiovascular, renal, neurological, and gastrointestinal system manifestations that contribute to morbidity and/or mortality, but render challenges to clinical trial endpoint selection. FDA representatives discussed regulatory perspectives regarding demonstration of clinical benefits of investigational therapies in the context of a rare disease with multi-systemic manifestations. The panel also highlighted the potential importance of well-designed health-related quality of life instruments, quantification of system organ effects, the potential of advanced imaging technologies, and survival prediction models.

**Conclusions:**

The Amyloidosis Forum identified a clear need for novel trial designs that are scientifically rigorous, feasible, and incorporate clinically meaningful endpoints based on an understanding of the natural history of the disease in an evolving therapeutic landscape. Future forums will delve into these issues and seek to include participation from additional stakeholders.

## Background

Immunoglobulin light chain (AL) amyloidosis (ORPHA:85443) is a rare disease caused by a monoclonal plasma cell disorder and characterized by the aggregation and deposition of insoluble amyloid fibrils derived from misfolding of monoclonal immunoglobulin light chains (LC). Despite its rarity, AL amyloidosis is the most commonly diagnosed systemic amyloidosis with an estimated prevalence of 1–5 per 10,000 [1] and an estimated incidence of 8–12 persons per million person-years [2, 3]. AL amyloidosis is multi-systemic and phenotypically heterogenous, affecting cardiac, renal, neurological, and gastrointestinal systems to varying degrees in different patients. Although there are no FDA approved treatments for AL amyloidosis, therapies that target clonal plasma cells are used off-label and are based on treatment paradigms established in multiple myeloma. There are no treatments specifically directed at correcting the amyloid fibril deposition that results in organ system dysfunction. Therefore, an unmet medical need persists despite advances in disease management. Collaboration and creative approaches are required to address the many barriers to development of effective therapies for this multi-systemic disease.

### AL amyloidosis public private partnership

**Fig. 1:**
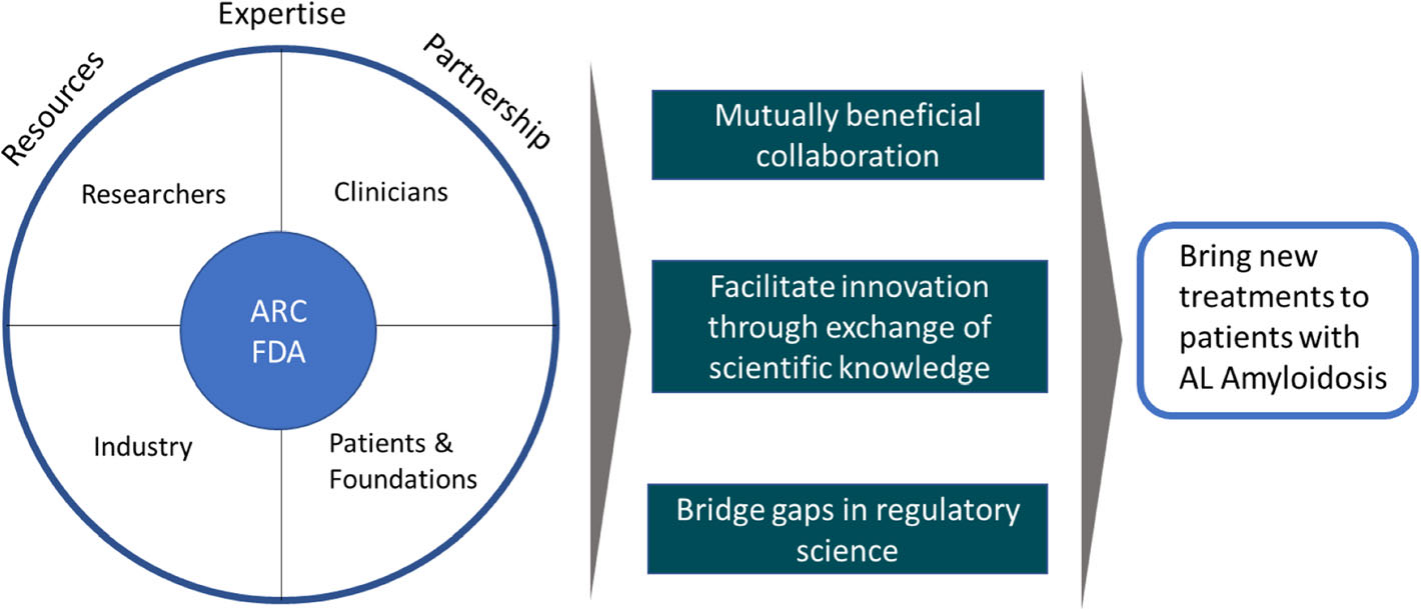
The Amyloidosis Forum Structure and Goals. The Amyloidosis Forum is a public-private partnership established to identify and bridge the scientific gaps in drug discovery and development for the treatment of AL Amyloidosis. The PPP seeks to leverage expertise and resources of all stakeholders (academia, industry, patients, and regulatory agencies) for the conduct of mutually beneficial scientific activities in the precompetitive domain to support bringing new, safe and efficacious therapies to patients with AL Amyloidosis

A public–private partnership (PPP) is a cooperative arrangement between two or more public and private sectors, typically of a long-term nature. In 2019, a PPP was formed between the nonprofit Amyloidosis Research Consortium (ARC; www.arci.org) and the US Food and Drug Administration (FDA) Center for Drug Evaluation and Research (CDER). The goal of this PPP is to identify and bridge the scientific gaps that are acting as barriers to drug discovery and development for the treatment of AL amyloidosis. The PPP seeks to leverage expertise and resources of all stakeholders (academia, industry, patients, and regulatory agencies) for the conduct of mutually beneficial scientific activities in the precompetitive domain to support bringing new, safe and efficacious therapies to patients with AL amyloidosis (Fig. 1).

To this end, CDER has appointed a liaison to the PPP who will inform participants about CDER’s regulation of drug products, provide its current thinking on precompetitive domain issues, provide general perspectives on the relative strength of the types of evidence that are present (or if there is a gap in the evidence), educate PPP participants on issues CDER reviewers are likely to consider important for precompetitive projects under development, and explain why certain issues may have importance to the CDER regulatory community. The CDER PPP liaison may arrange to bring other FDA staff into the discussion if needed. Importantly, CDER staff participation will not be related to any specific regulatory application, product, or other non-public information. Project results generated by this PPP will be made broadly available to the public to benefit public health.

This review summarizes proceedings of the first of an anticipated series of meetings convened at FDA on November 12, 2019. The inaugural Amyloidosis Forum focused on achieving a broad understanding of AL amyloidosis. The forum consisted of a series of presentations and panel discussions. The panel was comprised of 11 physicians from medical institutions in the US and Europe, 2 health outcomes professionals, representatives of ARC, 3 pharmaceutical companies, and 6 divisions of the FDA. Patients provided important perspectives on the path to diagnosis, challenges of rigorous treatment, and the burden of disease. The panel reviewed the epidemiology, pathobiology, and clinical features of AL amyloidosis. Hematological characteristics, staging systems, and response criteria were examined, and a clear consensus emerged that a “deep response” to plasma cell clone directed treatments was critical to OS. Emphasis was placed on the heterogeneous clinical phenotypes of AL amyloidosis, exemplified by varying impacts on the cardiovascular, renal, neurological, and gastrointestinal systems that all substantially affect morbidity. The heterogeneous presentations and disease courses make selection of endpoints for interventional clinical trials particularly challenging. FDA representatives discussed regulatory perspectives in the context of rare diseases and clinical outcomes assessments. The panel also highlighted the importance of patient-reported outcomes (PROs), the potential of imaging biomarkers to guide dose-selection, and the use of survival prediction models to guide trial length. The Amyloidosis Forum identified a clear need to identify, develop, and/or modify novel trial designs and clinical endpoints to encourage drug development in this rare disease. The availability of natural history data is crucially important to meet these needs.

### Path to diagnosis: myriad non-specific symptoms delay diagnosis and early access to clinical trials

**Fig. 2:**
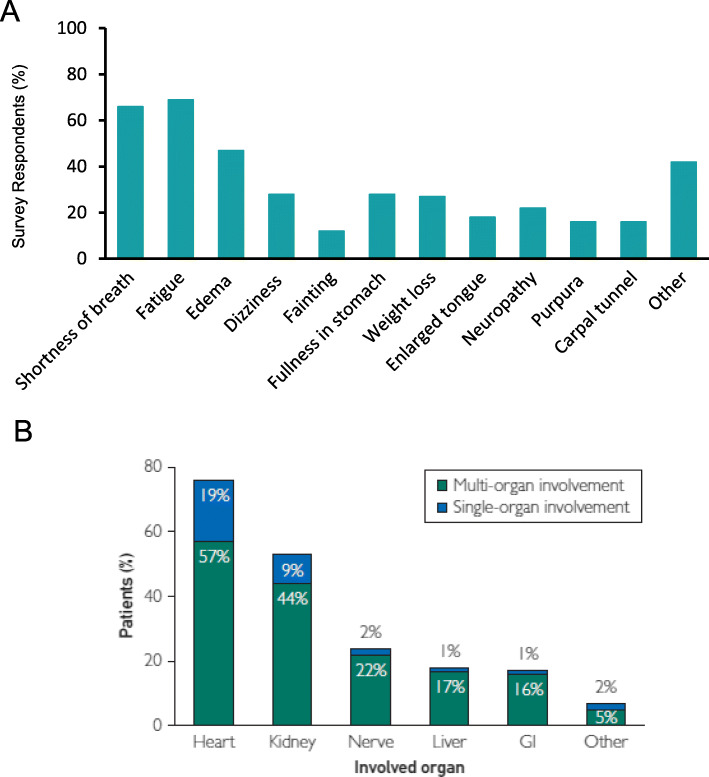
Prevalence of Presenting Symptoms and Organ Involvement. Most common presenting symptoms in AL amyloidosis patients based on global patient survey results (Panel **a**); adapted with permission from ARC. Organ involvement distribution (Panel **b**) in patients with mass-spectrometry-verified typing of AL amyloidosis (*N* = 592); reproduced with permission (Muchtar et al. Mayo Clin Proc. 2019;94 (3):472–483)

AL amyloidosis is sometimes termed a “great imitator disease.” Establishing an early and accurate diagnosis of amyloidosis was viewed as a significant challenge based on results of a global survey conducted in patients, family members, and caregivers [4]. Presenting symptoms vary widely depending on organ involvement and the extent of damage caused by amyloid deposits. The most common early symptoms are non-specific and include dyspnea, fatigue/weakness, palpitations, numbness, pain, altered bowl habits, and edema (Fig. 2a) leading most patients to be initially referred to cardiologists [4]. A majority of survey respondents (68.9%) saw 3 or more physicians prior to diagnosis; the time to accurate diagnosis exceeded 1 year for 37.1% of respondents. The diagnosis of AL amyloidosis was most commonly obtained from hematologist/oncologists (34.1%), followed by nephrologists (22.6%) and cardiologists (18.7%) [4]. New approaches are needed to identify AL patients earlier. The panel discussed how delays in diagnosis also have consequences for development of new treatment options as many AL amyloidosis patients are too sick to qualify for, or benefit from, participation in clinical trials with restrictive eligibility criteria.

## A multisystemic disease: pathobiology and clinical features confound targeted therapeutic approaches

Systemic AL amyloidosis is a monoclonal plasma cell disorder. More than 90% of patients have an abnormal serum free light chain ratio [5]. There are genomic differences in plasma cells between AL amyloidosis and multiple myeloma, but none that are specific to AL amyloidosis. For example, there is a notable overrepresentation of t(11;14) translocation in patients with AL amyloidosis compared to patients with multiple myeloma [6–10]. In AL amyloidosis, the mutational burden is limited; the variable antigen binding domains of the LC contribute to the targeting of specific organs. Conformational changes in the secondary or tertiary structure of the abnormal monoclonal LC induce abnormal folding and assembly of monomers that form amyloid fibrils [11]. In a multivariable analysis, the presence of ≥20% bone marrow plasma cells or AL CRAB (hypercalcemia, renal failure, anemia and bone disease) were independent risk factors for survival (Muchtar et al., unpublished). Patients with ≥20% plasma cells were also more likely to have cardiac involvement and other high-risk features (unfavorable cytogenetics, increased proliferative rate, CRAB features, immunoparesis).

Nearly all organs can be affected in systemic AL amyloidosis resulting in a myriad of clinical features; a majority of patients have involvement in one or two organs, with the heart (76%) and kidney (53%) being the most commonly impacted (Fig. 2b). Approximately 25% of patients have more than 2 involved organs [5]. Clinical presentation is based on target organ involvement and includes: cardiomyopathy (65%), nephrosis (65%), gastrointestinal symptoms (30%), peripheral neuropathy (20%), orthostasis (20%), hypothyroidism (19%), hepatomegaly (15%), macroglossia (12%), and carpal tunnel syndrome (10%) [11, 12]. Patient outcome is highly dependent on the spectrum and severity of organ involvement, especially for patients with cardiac involvement and/or progression to end stage renal disease (ESRD), which are the primary causes of mortality in patients with AL amyloidosis [13, 14].

To highlight the multi-systemic aspects of AL amyloidosis, the Forum panel included hematologist/oncologists, physicians who specialize in the treatment of the cardiac, renal, gastrointestinal, and neurological manifestations of the disease, and representatives from corresponding divisions of the FDA. The panel discussed specific comorbidities and challenges with cardiac, renal, gastrointestinal, and neurological involvement. Specifically, AL amyloidosis patients with kidney involvement are often asymptomatic; generally presenting with an abnormal glomerular filtration rate (GFR; 25%) and/or proteinuria (75%). Gastrointestinal involvement generally results in decreased absorption, depending on whether amyloid fibrils deposit in the mucosa or muscle/nerve. The most common gastrointestinal symptoms in AL amyloidosis are early satiety, weight loss, and constipation/diarrhea; all of which have a large impact on health-related quality of life (HRQOL). In patients with gastrointestinal involvement, signs and symptoms could potentially be collected directly from patients using a fit-for-purpose PRO instrument(s) to obtain a clinically meaningful measure of treatment benefit; however, these symptoms are often confounded by adverse drug effects and currently there is no fit-for-purpose AL amyloidosis-specific PRO instrument. The FDA encourages early discussion between sponsors and the review division on how to measure clinically relevant endpoints. Neurological involvement in AL amyloidosis may be autonomic (e.g. orthostatic intolerance, erectile dysfunction) and/or somatic (e.g. lack of feeling, foot drop). Composite neurological scores that incorporate the clinical, electrophysiological and autonomic attributes assessed by trained personnel are considered an appropriate measure of treatment response/regression.

Challenges for treating cardiac symptoms and assessing treatment response were also discussed by the panel. Many standard treatments for congestive heart failure (e.g. beta blockers, angiotensin converting enzyme [ACE] inhibitors, angiotensin receptor blocker [ARBs], and angiotensin receptor neprilysin inhibitors [ARNIs]) are either contraindicated or poorly tolerated at high doses in patients with AL amyloidosis. Discussion focused on the potential of emerging echocardiographic, magnetic resonance imaging (MRI), and nuclear/PET imaging techniques for providing objective measures of cardiac involvement and potentially standardized metrics for cardiac responses to therapies being evaluated in multicenter clinical trials. Imaging technology was identified as an area for further review by the Forum.

## Treatment modalities, response criteria, and longitudinal outcomes

There has been an evolution of AL amyloidosis staging systems based on variables independently prognostic for OS. Disease staging is driven by factors influencing prognosis (i.e. extent of cardiac and/or other organ involvement), and the “tumor” burden/characteristics. In AL amyloidosis, response assessment includes both hematologic response and involved organ response. Published literature suggests a correlation between the depth of hematologic response and improved OS [10, 15–19]. Emerging data suggest that achieving a minimal residual disease negative state in the bone marrow is associated with progression-free survival [18, 20–23]. The panel agreed that the primary initial goal of treatment is to reduce/eliminate the LC burden before organ damage occurs and to allow for organ healing once it has occurred.

Although there are no FDA approved therapies for the treatment of AL amyloidosis, treatment modalities for AL amyloidosis tend to follow multiple myeloma treatment paradigms. High-dose melphalan followed by autologous stem cell transplant (HDM-SCT) is among the most effective cytotoxic therapies against plasma cells and leads to reduced production of the abnormal LC and prolonged survival. However only 25–35% of patients with AL amyloidosis are eligible for HDM-SCT because of poor functional status, advanced cardiac involvement, advanced renal dysfunction, low blood pressure due to autonomic neuropathy, or age [24, 25]. Non-SCT first-line regimens, including cyclophosphamide, dexamethasone, and bortezomib-based therapies, have changed dramatically over the past 20 years and continue to evolve as advancements are made in the availability of new agents for the treatment of multiple myeloma. Overall, outcomes have improved over the past 20 years with earlier diagnosis, higher rates of very good partial response (VGPR), lower early mortality, and improved OS [21, 26]. Currently, median OS for patients with AL amyloidosis is approximately 6 years, with 22% of patients alive at 10 years; nearly 50% of 10-year survivors had only one line of therapy [3, 21].

Predictors of organ response include the severity of organ dysfunction at diagnosis, time to treatment, plasma cell burden, and the depth of hematological response. The number of organs with a response following treatment (i.e. all major organs, at least one organ, or no organ response) correlates with OS [10]. The depth of the organ response in AL amyloidosis is also associated with improved OS [21]. Response criteria have been proposed for patients with renal involvement based on combinations of proteinuria reduction and eGFR. Renal response 6 months following treatment has some predictive utility for progression to dialysis at 2-years and 5-years [27]. Graded response (and progression) criteria for heart (NT-proBNP), kidney (proteinuria), and liver (alkaline phosphatase, AP) involvement have also been proposed [21].

**Fig. 3:**
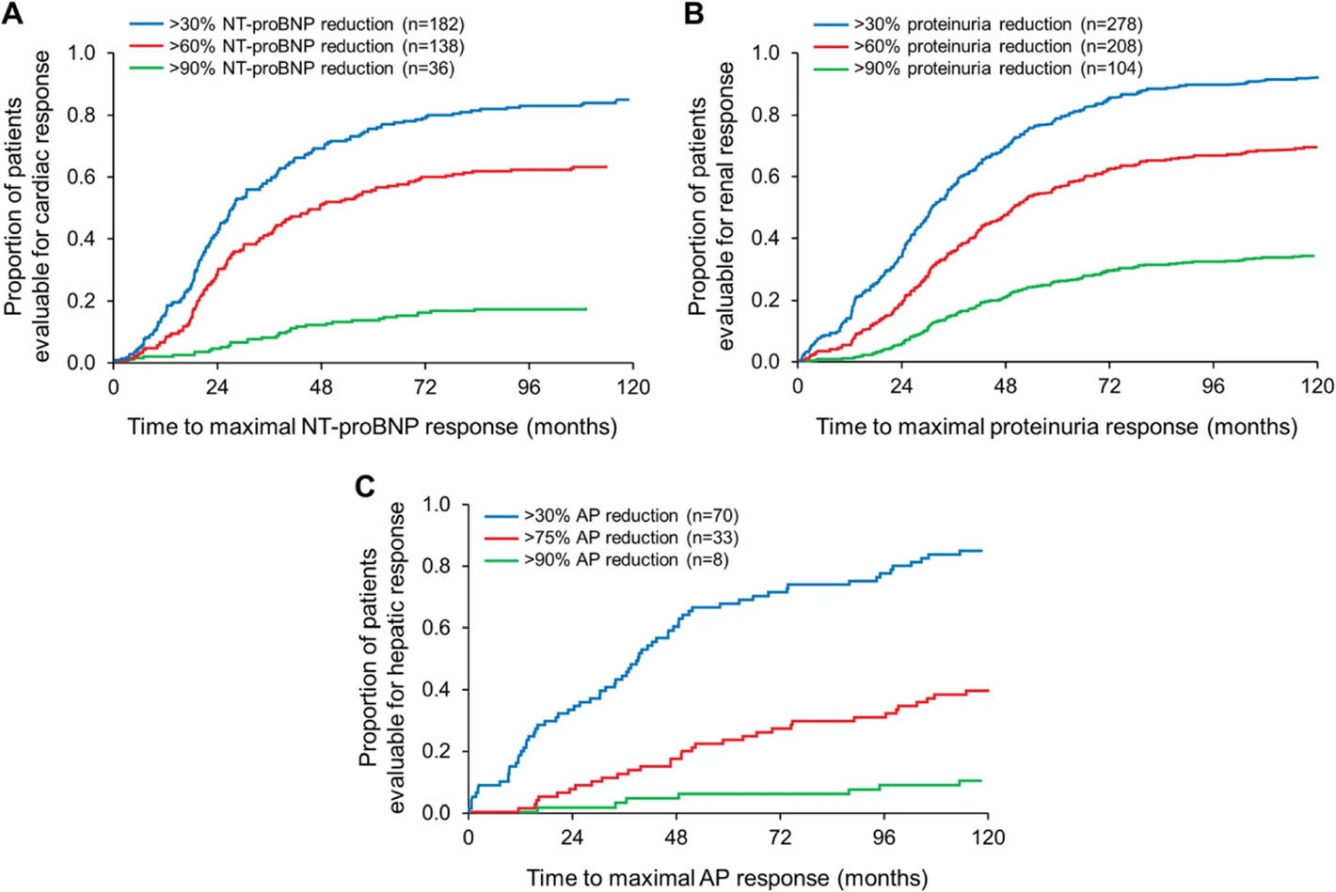
Kinetics of Organ Response. Time to achievement of maximal organ response stratified by increased order of depth of organ response in patients with cardiac (Panel **a**; NT-proBNP response), renal (Panel **b**; proteinuria response), or hepatic (Panel **c**; alkaline phosphate response) involvement. Patients (*N* = 414) were included if they achieved either a hematologic or organ response. Reproduced with permission (Muchtar et al. Leukemia. 2018;32 (10):2240–2249)

The kinetics of hematologic and organ responses are distinctly different, with organ responses being delayed following hematologic response. Initial organ responses are observed at 6–9 months post treatment initiation with maximal responses observed between 24 and 36 months post treatment initiation (Fig. 3) [23, 28]. The discordant kinetics of the hematologic and organ responses is a critical issue for clinical care and in clinical trial design, specifically with regard to decisions of when to add, switch, or resume therapy based on hematologic and/or organ responses.

Although hematologic response is necessary for organ response, it may not be sufficient because organs may deteriorate despite hematologic response. Possible reasons for this include irreversible organ damage at diagnosis and/or low-level pathogenic LCs produced by minimal residual clonal disease. For example, in patients with advanced renal involvement, even a deep hematologic response may not be sufficient to prevent the development of ESRD. In this population, hematologic responses are difficult to determine because serum free light chain (FLC) clearance is dependent on renal function.

**Fig. 4:**
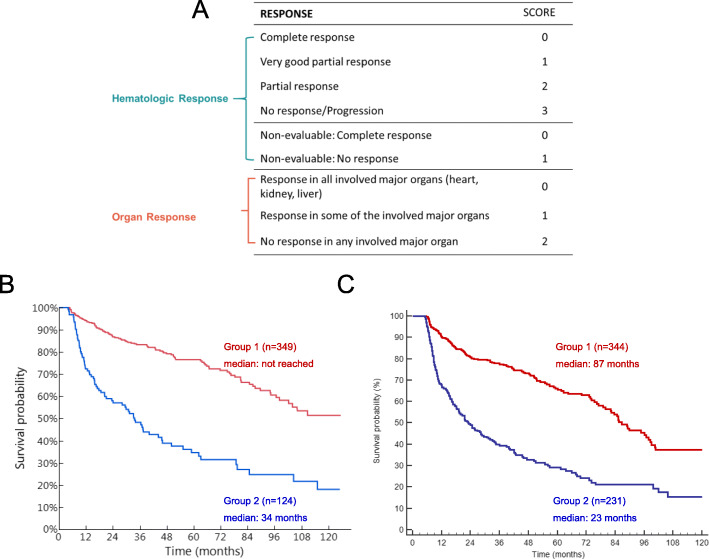
Overall Survival by Composite Organ and Hematologic Response. The composite hematologic and organ response (CHOR) model (Panel **a**). Group 1 defined by summary score of 0–3; Group 2 defined by summary score of 4–5. Overall survival in the composite model was similar in the Mayo Clinic (Panel **b**; *p* < 0.001) and Pavia (Panel **c**; *p* < 0.001) cohorts. Reproduced with permission (Sidana et al. 2017. Am Soc Hematol. 130:abstract #3046)

The development of composite response criteria that address both the hematologic and organ responses may provide a new outcome measure to assess both the underlying plasma cell dyscrasia and the resulting organ damage inherent to AL amyloidosis. A proposed composite index would rank established hematologic criteria and separately rank organ response. Initial analysis conducted on two separate cohorts demonstrated a significant difference in survival probability between two groups based on composite scores; results were reproduced in both cohorts (Fig. 4) [10]. Validation of organ and composite response criteria are ongoing.

Overall, patient prognosis is dependent on many factors: the extent of cardiac involvement and/or other organ involvement, “tumor” burden, the depth of hematologic response and organ response. For patients with AL amyloidosis, the extent of cardiac involvement drives OS and presents the greatest unmet medical need.

## Burden of illness: detecting clinically meaningful impacts on health-related quality of life

During the Forum, three patients shared their stories and highlighted the varying degree to which AL amyloidosis is a multi-systemic disease posing numerous challenges. Patients provided important perspectives on the path to diagnosis, challenges of rigorous treatment, and the burden of disease. These important testimonials shared commonality: AL amyloidosis and current treatment both significantly and broadly impact HRQOL. Treatment has a major impact on HRQOL because the rigorous therapeutic regimens are difficult for patients to tolerate. Published reports and qualitative interviews with physicians and patients have further characterized the burden of disease in AL amyloidosis [4, 29–31].

A conceptual model of AL amyloidosis was developed to depict the impact of disease, treatment, and relationships among the impacts [30]. The conceptual model provided an overall picture of the symptoms associated with AL amyloidosis by organ involvement, treatment impacts on patient functioning and well-being; and was based upon a disease model that shows how the proximal impacts of disease influence patient functioning and HRQOL [32]. As depicted in the model, the symptoms associated with AL amyloidosis are numerous and variable. The impact of AL amyloidosis on HRQOL ranges from impairment of physical function to emotional distress, including impairments in mobility, work, sleep, participation in family activities and social relationships, and mental health functioning.

Several PRO instruments have been utilized as outcome measures in AL amyloidosis studies, including organ-specific measures (Kansas City Cardiomyopathy Questionnaire, Norfolk Quality of Life Questionnaire for diabetic neuropathy), health utility scores (EQ-5D, SF-6D), and generic measures of health status (SF-36-v2, Hematology Patient Reported Symptom Screen). One of the health status measures discussed at the Forum was the SF-36, a PRO instrument that has been widely used in clinical studies [31]. The psychometric properties of SF-36 in patients with AL amyloidosis have been assessed using data from community-based (*n* = 341) and clinic-based (*n* = 1438) observational studies [33].

**Fig. 5:**
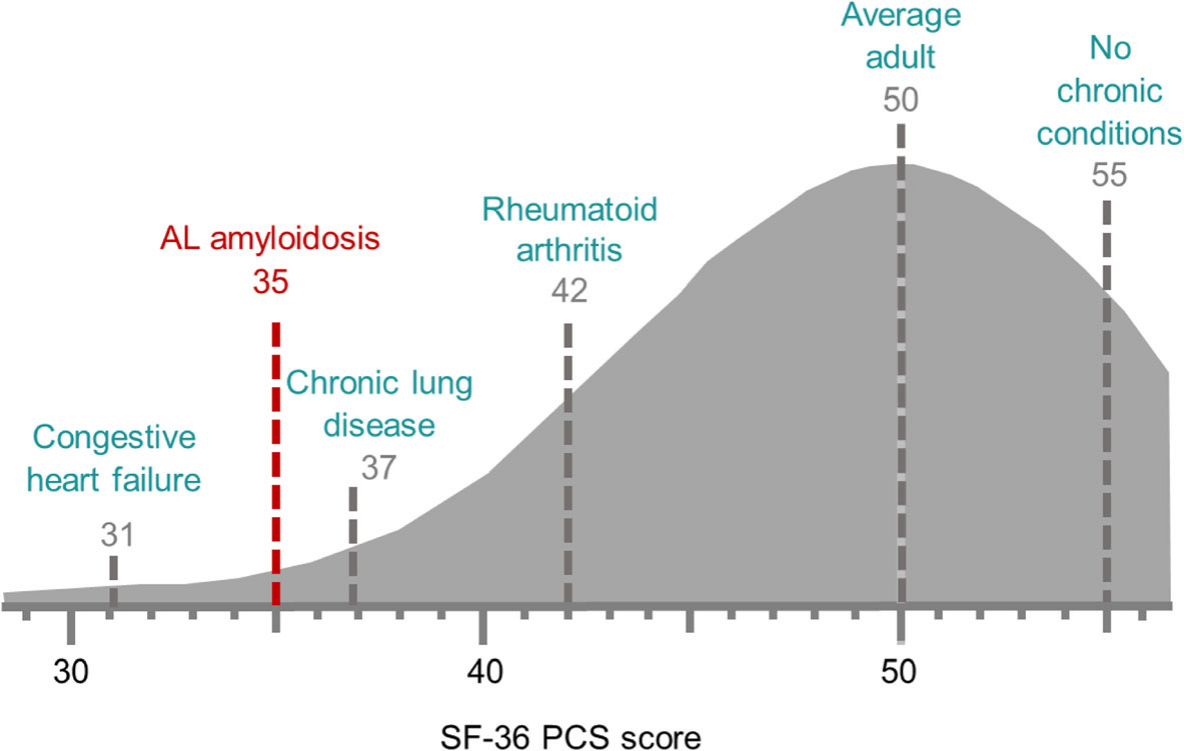
SF-36 Benchmark Scores. SF-35 physical component summary (PCS) score of AL amyloidosis patients relative to other chronic conditions in the US population. Reproduced with permission (Sanchorawala et al. Oral presentation at: Amyloidosis Research Consortium Key Opinion Leader Meeting; September 16, 2015; Boston MA)

The SF-36 has also been used to document the general burden of disease in AL amyloidosis in a cross-sectional, observational study [33] and in a longitudinal analysis following HDM-SCT treatment [34]. Both studies showed much lower scores in all domains of the SF-36 relative to the average score for an adult in the US. In the longitudinal analysis of HRQOL following HDM-SCT (*N* = 544), patients with AL amyloidosis averaged a baseline physical component summary (PCS) score of 35; significantly lower than age-matched population norms (Fig. 5). For perspective, this lies in a severity spectrum similar to patients with congestive heart failure (PCS = 31) or chronic lung disease (PCS = 37). Clinical characteristics associated with reduced PCS included performance status, neuropathy, gastrointestinal/liver disease, and weight loss. Clinical characteristics associated with reduced mental component summary (MCS) scores included neuropathy, weight loss and performance status. Following treatment, SF-36 scores improved; MCS reached the population norm 1-year post HDM-SCT and PCS reached the population norm 2 years post HDM-SCT [34]. Levels of cardiac biomarkers (NT-proBNP) correlated with mean SF-36 score; an association between risk of death and baseline SF-36 PCS scores has also been shown [35]. SF-36 scores were informative when examining factors associated with early post-treatment survival and subsequent survival beyond 1-year follow-up.

The challenges of the temporal disconnect between a drug exhibiting its activity, potential worsening of symptoms due to treatment effects, and the timing of detecting clinically meaningful improvements in HRQOL for the AL amyloidosis population were also identified as critical issues. The panel discussed the importance of PROs as one type of clinical outcomes assessment in AL amyloidosis trials. The FDA encourages the use of fit-for-purpose PROs in all drug development programs that reflect the concept of interest. Prospective instrument development studies and/or data from natural history studies and Phase 1/2 trials may be used to develop and test specific fit-for-purpose PRO measures.

## FDA approach to multi-systemic diseases

Because AL amyloidosis is a rare, multisystemic disease, development of experimental treatments may involve different divisions of the FDA and requires a multidisciplinary approach. To this end, panel representatives from the Divisions of Hematologic Malignancies II; Gastroenterology and Inborn Errors Products; Cardiovascular and Renal Products (cardiologist and nephrologist); Neurology Products, the Rare Diseases Program, and Clinical Outcomes Assessments Staff each provided perspectives on the Agency’s approach to multi-systemic diseases. The breadth of engagement and involvement across several divisions provided a unique opportunity for attendees and panel members. The representatives all recognized the importance and challenge of drug development for rare, heterogeneous diseases such as AL amyloidosis and emphasized the need for a collaborative, multi-disciplinary approach to drug development. Engagement of regulatory agencies by both industry and patient advocacy groups was encouraged “early and often,” beginning as early as the nonclinical development phase.

The FDA discussed both traditional and accelerated approval pathways and considerations for choosing appropriate primary endpoints. In the traditional approval pathway, substantial evidence of efficacy can be established by demonstration of improvement of a clinically meaningful endpoint (e.g. a direct measure of how a patient feels, functions, or survives) or a validated surrogate endpoint (an endpoint that is not a direct measure of clinical benefit but for which there is strong evidence that it predicts benefit). Approval requires successful conduct of two adequate and well-controlled trials. In special circumstances, when it is highly impracticable to conduct two trials, the successful conduct of one adequate and well-controlled trial with confirmatory evidence (e.g. pharmacodynamic data from other studies that would likely predict the effect on the demonstrated clinical endpoint based on what is known about the disease pathophysiology) may be sufficient to support approval.

Several expedited programs may help accelerate drug approval for rare diseases that are serious and life-threatening. The accelerated approval program is a pathway to marketing approval for a new drug product on the basis of adequate and well-controlled clinical trials that establish an effect on a surrogate or intermediate endpoint that is reasonably likely, based on epidemiologic, therapeutic, pathophysiologic, or other evidence, to predict a meaningful clinical benefit over existing treatments on the life-threatening or severely debilitating aspects of the condition. Post-marketing confirmatory trials may be required to verify and describe the anticipated effect. The FDA panelists highlighted the difficulties with use of biomarkers that are presumed to be surrogates, citing the variability of NT-proBNP changes from baseline with mortality outcomes in clinical trials, the experience with ventricular arrhythmia suppression in the CAST trial, and the increased mortality that has been seen with cardiac inotropes in spite of short term hemodynamic improvements. Published evidence in AL amyloidosis suggests NT-proBNP levels are associated with cardiac function and OS in AL amyloidosis [36, 37]. It has been postulated that NT-proBNP levels in AL amyloidosis reflect organ damage due to direct insult to ventricular cardiomyocytes by toxic light chains or amyloid fibrils as opposed to non-AL amyloid heart failure in which multiple mechanisms contribute to NT-proBNP levels [38].

The feasibility of a particular endpoint is dependent on an understanding of the natural history of disease progression, mechanism of action of the drug, and the ability to identify the population in which the treatment benefit can be detected. Specific challenges were acknowledged for investigating AL amyloidosis, including the heterogeneity of the disease (which may necessitate novel or adaptive trial designs), the need for tailored statistical approaches and focused trial populations, and the process for development of novel clinical outcomes to measure and predict responses. Given the limited populations available for study of rare diseases and the importance of patient-focused drug development, the FDA has issued a series of guidance documents intended to assist pharmaceutical companies and patient groups in the design of trials with clinical outcomes that reflect the patient voice and clinical benefit, and that are sensitive enough to detect meaningful changes (https://www.fda.gov/drugs/development-approval-process-drugs/fda-patient-focused-drug-development-guidance-series-enhancing-incorporation-patients-voice-medical).

The concept of patient-informed drug development was a key theme in the development of new therapies to maximize benefit. In this context, natural history data are viewed as central to defining endpoints, developing PROs and other clinical outcomes measures, understanding disease progression, and to informing design of interventional trials. Retrospective data from amyloidosis databases at academic institutions and data from control arms of industry-sponsored interventional trials are potential sources of valuable natural history data. A collaborative approach to obtaining natural history data between industry, regulatory agencies, and patient advocacy groups has the potential to expedite development of new clinical outcomes measures and/or modification of existing instruments.

The utility of retrospective natural history data and its potential use as a historical control in a clinical trial were also discussed. For AL amyloidosis, natural history data that preceded the availability of important additions to plasma cell reduction therapies may be of limited utility as a historical control in trials of clinical outcomes and/or specific organ system responses. Therefore, prospective controlled trials are optimal, although they pose additional challenges in the absence of standard care in AL amyloidosis.

Discussion among panelists highlighted these and several other challenges to drug development for AL amyloidosis, including delays to drug development while conducting prospective natural history studies, reliance on retrospective data in a changing anti-plasma cell treatment landscape, and assumptions that efficacy and/or safety in one organ system can be extrapolated to all other organ systems. While the Agency representatives acknowledged limited experience with composite endpoints and hierarchical ranked sum analyses that include components spanning cardiovascular, renal, neurological, gastrointestinal, and hematologic organ systems, a multiple endpoint concept may be helpful for heterogeneous diseases such as AL amyloidosis (https://www.fda.gov/media/102657/download). When considering a multiple endpoint concept, careful consideration should be given to identifying the endpoint components (and the magnitude of the changes of those components) that would represent clinically meaningful effects.

## Challenges in trial design: endpoints

**Fig. 6:**
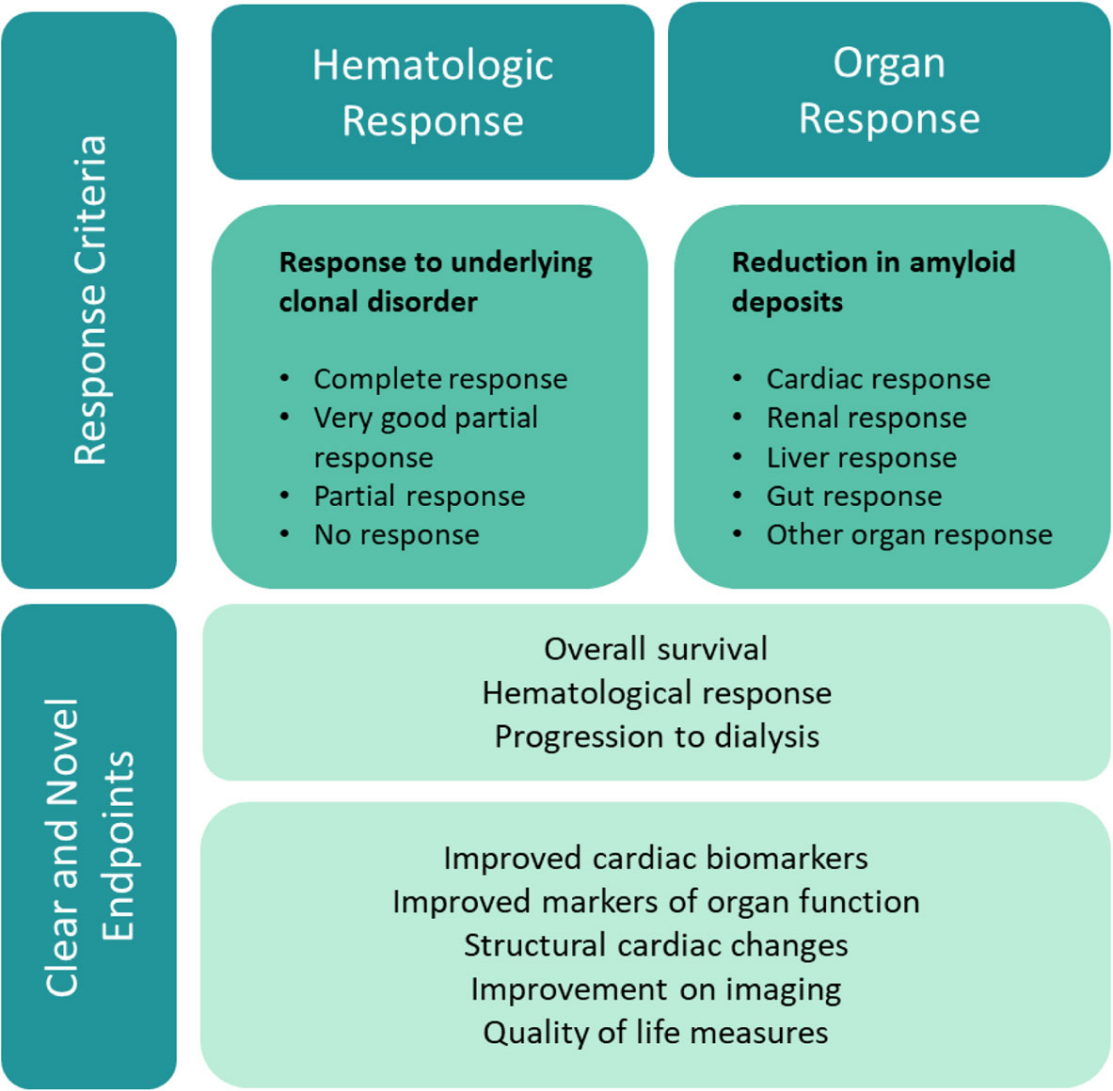
Clinical Trial Endpoints. Overview of established hematologic response criteria and target organs to measure responses to treatment in relation to established objective endpoints and the identified need for novel endpoints to enable early and robust trial readouts

Treatments for AL amyloidosis generally either target the cause (i.e. clonal plasma cell production of excess serum FLC) or the downstream effect (i.e. amyloid deposition, organ damage, and progressive organ failure). Selection of trial endpoints must therefore consider the drug’s intended mechanism of action. Chemotherapy, HDM-SCT, immunotherapy and other investigational agents that target the underlying clonal disorder should assess the hematological response. Therapeutic approaches targeting amyloid deposition and organ function (e.g. agents to accelerate clearance, reduce tissue toxicity, or protein stabilizers) should assess organ response(s) (e.g. heart, kidney, gut, peripheral nerves) and ensure there is no worsening of clinical outcomes. Currently, only anti-plasma cell therapies have demonstrated clinical benefit and there remains much to learn about assessment of “anti-amyloid” treatments (Fig. 6).

Objective endpoints of OS and hematologic responses are primary endpoints with clear clinical benefit. However, depending on the disease severity of the studied population, OS response analyses may require trials of extended duration, obligating patients and resources that might be better used for the development of a different therapy. Potential pharmacodynamic endpoints for phase 2 studies could include laboratory and/or imaging biomarkers, although many (non-renal) biomarkers have limitations for regulatory acceptance as primary endpoints in phase 3 trials. There was consensus on the need to capture symptom responses inherent to AL amyloidosis and the development of fit-for-purpose PROs (Fig. 6). Functional outcomes, including the 6-min walk test, are challenging for the AL amyloidosis population given disease heterogeneity, near-term worsening due to adverse treatment effects, and the kinetics of delayed organ response [39]. More work is needed to establish improved markers of organ function and improved imaging modalities to assess structural cardiac changes (e.g. MRI extracellular volume, global longitudinal strain) and peripheral organ involvement to provide early and robust trial readouts.

Given the heterogeneity of the disease, trial populations may be subgrouped by organ involvement. The extent of cardiac involvement has a large impact on OS and therefore represents a patient population with unmet medical need and a clear objective primary endpoint for interventional trials. For advanced patients with severe cardiac involvement (Mayo Stage IIIb), improvement in OS is an achievable endpoint. However, for this severely affected cardiac population, anti-plasma cell treatment may not directly lead to end organ improvement; therefore high mortality due to disease progression should be anticipated by Institutional Review Boards/Independent Ethics Committees, sponsors, and regulators. There also may be difficulty in distinguishing toxicity due to investigational product from symptoms of disease. Endpoint selection in patients with early (Mayo Stage II) and moderate (Mayo Stage IIIa) cardiac involvement are more challenging.

Patients with renal involvement have a high risk of progression to ESRD; it may be possible to design a composite endpoint based on eGFR, proteinuria, and the need for renal replacement therapy to detect a benefit in this population [27]. If patients are symptomatic from renal disease (e.g., related to marked proteinuria), it may also be possible to develop endpoints to assess improvements in those symptoms.

## Challenges in trial design: patient populations

Selection of an appropriate study population is paramount to successful trial design; however, AL amyloidosis trials present several limitations. Eligibility criteria that are too selective may restrict the population of an already rare, heterogeneous disease and pose challenges for enrollment and subsequent development of treatments to benefit all AL amyloidosis patients. Unnecessarily restrictive eligibility criteria may also limit safety data in important subgroups, such as those with renal involvement and reduced kidney function.

While there are challenges in designing trials with newly diagnosed patients, the study of patients with relapsed/refractory AL amyloidosis presents additional challenges. Established response criteria, median OS, cardiac and renal response/progression criteria were all defined using reference data from the first-line therapy setting [27, 37] and may not be applicable to second- or third-line therapy in relapsed/refractory patients. Hematologic progression criteria from a complete response (CR) were initially defined as any detectable monoclonal protein or an abnormal FLC ratio. Progression from a partial response PR was defined as a 50% increase in serum or urine m-protein to > 0.5 g/dL or 200 mg/d respectively; or a 50% increase in FLC to > 10 mg/dL [40]. These progression criteria are largely viewed as insufficient given that the level of FLC that causes downstream organ toxicity has not been defined. It is not known if FLC accurately assesses the burden of hematologic disease in relapsed patients or whether more sensitive measures of hematologic disease would allow for earlier treatment and possibly organ preservation.

A recent study indicated 35% of patients (92/259) who responded to first-line therapy required second-line treatment after a median of 49 months; of the 92 patients, 48% did not have “measurable disease” [41]. Patients with hematologic and/or organ progression (relapsed/refractory) are often ineligible for salvage therapy trials based on the requirement for a difference between involved and uninvolved free light chains (dFLC) > 5 mg/dL. Patients with organ progression in the absence of hematologic progression also are often ineligible. Similar challenges exist in designing trials for patients with AL amyloidosis in both the first-line and relapsed/refractory setting with notable exceptions including longer OS, an increased number of patients without “measurable disease,” increased heterogeneity in organ involvement, a lack of validated hematologic response/progression criteria, and no validated organ response/progression criteria for relapsed patients. “Traditional” hematologic response criteria may need to be modified for this population using stringent dFLC criteria (< 1 mg/dL).

The panel further highlighted an inherent disconnect between the speed of clinical trial enrollment and the need to provide relapsed/refractory patients access to clinical trials. Physicians are also hesitant to wait to treat patients until stringent criteria for relapse are met; an issue further confounded by the delayed kinetics of organ response to first line therapy. The panel discussed whether a VGPR is “good enough” or whether additional lines of therapy should be introduced sooner given that the balance of FLC and organ deposition is still poorly understood. Overall, the current climate does not provide incentive for sponsors to design salvage trials since they often take years to accrue patients and demonstration of improved OS may not be easily achieved. At the same time, symptom scores and HRQOL PROs are likely not robust enough or applicable in the relapsed/refractory setting. Potentially, development of a composite or multi-domain responder index may be helpful for drug development in the relapsed/refractory setting provided it can be developed based on robust understanding of natural history and available Phase 1/2 data.

## Conclusions - Areas for Future Investigation to Address Unmet Needs

The inaugural Amyloidosis Forum provided a basic disease primer and identified challenges and opportunities in the development of new treatments to decrease the mortality, improve function, and/or improve HRQOL in patients with AL amyloidosis. The Forum identified a clear need for novel trial designs and clinical endpoints to address the burden of disease and therapeutic effects, the development of which necessitates natural history data in an evolving therapeutic landscape. Development of PROs tailored to symptomatic burden and HRQOL in AL amyloidosis may be required to provide specificity of therapeutic effect and inform mechanism of action and to measure burden of illness during therapy. Qualification of robust and reproducible imaging techniques may provide objective clinical outcomes to assess burden of disease and response to therapy. Future forums will delve into these issues and seek to include participation from additional stakeholders. The PPP is envisioned to serve as a model approach to address challenges in precompetitive drug development for other rare diseases.

## Data Availability

The information and data presented in this publication were presented at the Inaugural Amyloidosis Forum, US Food and Drug Administration White Oak Campus on 12 November 2019. Presentations are publicly available at: https://amyloidosisforum.org

## Abbreviations

AL or LC: Immunoglobulin light chain
ARC: Amyloidosis Research Consortium
CDER: Center for Drug Evaluation and Research
CR: Complete response
CRAB: Hypercalcemia, renal failure, anemia and bone disease
eGFR: Estimated glomerular filtration rate
ESRD: End stage renal disease
FDA: United States Food and Drug Administration
FLC: Free light chain
dFLC: Difference between serum involved and uninvolved free light chain
HDM-SCT: High-dose melphalan/autologous stem cell transplant
HRQOL: Health related quality of life
MCS: Mental component summary
MRI: Magnetic resonance imaging
NT-proBNP: N-terminal prohormone of brain natriuretic peptide
OS: Overall survival
PCS: Physical component summary
PPP: Public private partnership
PRO: Patient reported outcome
VGPR: Very good partial response

## References

[1] Merlini G, Comenzo RL, Seldin DC, Wechalekar A, Gertz MA (2014). Immunoglobulin light chain amyloidosis. Expert Rev Hematol.

[2] Kyle RA, Larson DR, Kurtin PJ, Kumar S, Cerhan JR, Therneau TM (2019). Incidence of AL amyloidosis in Olmsted County, Minnesota, 1990 through 2015. Mayo Clin Proc.

[3] Merlini G, Dispenzieri A, Sanchorawala V, Schonland SO, Palladini G, Hawkins PN (2018). Systemic immunoglobulin light chain amyloidosis. Nat Rev Dis Primers.

[4] Lousada I, Comenzo RL, Landau H, Guthrie S, Merlini G (2015). Light chain amyloidosis: patient experience survey from the amyloidosis research consortium. Adv Ther.

[5] Muchtar E, Gertz MA, Kyle RA, Lacy MQ, Dingli D, Leung N (2019). A modern primer on light chain amyloidosis in 592 patients with mass spectrometry-verified typing. Mayo Clin Proc.

[6] Merlini G, Stone MJ (2006). Dangerous small B-cell clones. Blood.

[7] Paiva B, Martinez-Lopez J, Corchete LA, Sanchez-Vega B, Rapado I, Puig N (2016). Phenotypic, transcriptomic, and genomic features of clonal plasma cells in light-chain amyloidosis. Blood.

[8] Meziane I, Huhn S, Filho M, Weinhold N, Campo C, Nickel J (2017). Genome-wide association study of clinical parameters in immunoglobulin light chain amyloidosis in three patient cohorts. Haematologica.

[9] Sidiqi MH, Aljama MA, Muchtar E, Buadi FK, Warsame R, Lacy MQ (2018). Light chain type predicts organ involvement and survival in AL amyloidosis patients receiving stem cell transplantation. Blood Adv.

[10] Sidana S, Tandon N, Dispenzieri A, Gertz MA, Buadi FK, Lacy MQ (2018). Clinical presentation and outcomes in light chain amyloidosis patients with non-evaluable serum free light chains. Leukemia.

[11] Desport E, Bridoux F, Sirac C, Delbes S, Bender S, Fernandez B (2012). Al amyloidosis. Orphanet J Rare Dis.

[12] Chaulagain CP, Comenzo RL (2015). How we treat systemic light-chain amyloidosis. Clin Adv Hematol Oncol.

[13] Kyle RA, Greipp PR, O'Fallon WM (1986). Primary systemic amyloidosis: multivariate analysis for prognostic factors in 168 cases. Blood.

[14] Kumar S, Dispenzieri A, Lacy MQ, Hayman SR, Buadi FK, Colby C (2012). Revised prognostic staging system for light chain amyloidosis incorporating cardiac biomarkers and serum free light chain measurements. J Clin Oncol.

[15] Dispenzieri A, Lacy MQ, Katzmann JA, Rajkumar SV, Abraham RS, Hayman SR (2006). Absolute values of immunoglobulin free light chains are prognostic in patients with primary systemic amyloidosis undergoing peripheral blood stem cell transplantation. Blood.

[16] Dittrich T, Bochtler T, Kimmich C, Becker N, Jauch A, Goldschmidt H (2017). AL amyloidosis patients with low amyloidogenic free light chain levels at first diagnosis have an excellent prognosis. Blood.

[17] Milani P, Basset M, Russo F, Foli A, Merlini G, Palladini G (2017). Patients with light-chain amyloidosis and low free light-chain burden have distinct clinical features and outcome. Blood.

[18] Tandon N, Sidana S, Dispenzieri A, Gertz MA, Lacy MQ, Dingli D (2018). Impact of involved free light chain (FLC) levels in patients achieving normal FLC ratio after initial therapy in light chain amyloidosis (AL). Am J Hematol.

[19] Manwani R, Cohen O, Sharpley F, Mahmood S, Sachchithanantham S, Foard D (2019). A prospective observational study of 915 patients with systemic AL amyloidosis treated with upfront bortezomib. Blood.

[20] Palladini G, Merlini G (2016). What is new in diagnosis and management of light chain amyloidosis?. Blood.

[21] Muchtar E, Gertz MA, Kumar SK, Lacy MQ, Dingli D, Buadi FK (2017). Improved outcomes for newly diagnosed AL amyloidosis between 2000 and 2014: cracking the glass ceiling of early death. Blood.

[22] Kastritis E, Kostopoulos IV, Terpos E, Paiva B, Fotiou D, Gavriatopoulou M (2018). Evaluation of minimal residual disease using next-generation flow cytometry in patients with AL amyloidosis. Blood Cancer J.

[23] Sidiqi MH, Aljama MA, Jevremovic D, Muchtar E, Buadi FK, Warsame R (2018). Prognostic significance of stringent complete response after stem cell transplantation in immunoglobulin light chain amyloidosis. Biol Blood Marrow Transplant.

[24] Sidiqi MH, Aljama MA, Buadi FK, Warsame RM, Lacy MQ, Dispenzieri A (2018). Stem cell transplantation for light chain amyloidosis: decreased early mortality over time. J Clin Oncol.

[25] Varga C, Comenzo RL (2019). High-dose melphalan and stem cell transplantation in systemic AL amyloidosis in the era of novel anti-plasma cell therapy: a comprehensive review. Bone Marrow Transplant.

[26] Jaccard A, Comenzo RL, Hari P, Hawkins PN, Roussel M, Morel P (2014). Efficacy of bortezomib, cyclophosphamide and dexamethasone in treatment-naive patients with high-risk cardiac AL amyloidosis (Mayo Clinic stage III). Haematologica.

[27] Palladini G, Hegenbart U, Milani P, Kimmich C, Foli A, Ho AD (2014). A staging system for renal outcome and early markers of renal response to chemotherapy in AL amyloidosis. Blood.

[28] Kaufman GP, Dispenzieri A, Gertz MA, Lacy MQ, Buadi FK, Hayman SR (2015). Kinetics of organ response and survival following normalization of the serum free light chain ratio in AL amyloidosis. Am J Hematol.

[29] McCausland KL, White MK, Guthrie SD, Quock T, Finkel M, Lousada I (2018). Light chain (AL) amyloidosis: the journey to diagnosis. Patient.

[30] Lin HM, Seldin D, Hui AM, Berg D, Dietrich CN, Flood E (2015). The patient's perspective on the symptom and everyday life impact of AL amyloidosis. Amyloid.

[31] Bayliss M, Rendas-Baum R, White MK, Maruish M, Bjorner J, Tunis SL (2012). Health-related quality of life (HRQL) for individuals with self-reported chronic physical and/or mental health conditions: panel survey of an adult sample in the United States. Health Qual Life Outcomes.

[32] Wilson IB, Cleary PD (1995). Linking clinical variables with health-related quality of life. A conceptual model of patient outcomes. JAMA.

[33] White MK, McCausland KL, Sanchorawala V, Guthrie SD, Bayliss MS (2017). Psychometric validation of the SF-36 health survey in light chain amyloidosis: results from community-based and clinic-based samples. Patient Relat Outcome Meas.

[34] Seldin DC, Anderson JJ, Sanchorawala V, Malek K, Wright DG, Quillen K (2004). Improvement in quality of life of patients with AL amyloidosis treated with high-dose melphalan and autologous stem cell transplantation. Blood.

[35] Sanchorawala V, McCausland KL, White MK, Bayliss MS, Guthrie SD, Lo S (2017). A longitudinal evaluation of health-related quality of life in patients with AL amyloidosis: associations with health outcomes over time. Br J Haematol.

[36] Merlini G, Lousada I, Ando Y, Dispenzieri A, Gertz MA, Grogan M, et al. Rationale, application and clinical qualification for NT-proBNP as a surrogate end point in pivotal clinical trials in patients with AL amyloidosis. Leukemia. 2016;30(10):1979–86. 10.1038/leu.2016.191 PMC5056962 27416985

[37] Palladini G, Dispenzieri A, Gertz MA, Kumar S, Wechalekar A, Hawkins PN (2012). New criteria for response to treatment in immunoglobulin light chain amyloidosis based on free light chain measurement and cardiac biomarkers: impact on survival outcomes. J Clin Oncol.

[38] Maurer MS, Elliott P, Comenzo R, Semigran M, Rapezzi C (2017). Addressing common questions encountered in the diagnosis and Management of Cardiac Amyloidosis. Circulation.

[39] Manwani R, Foard D, Mahmood S, Sachchithanantham S, Lane T, Quarta C (2018). Rapid hematologic responses improve outcomes in patients with very advanced (stage IIIb) cardiac immunoglobulin light chain amyloidosis. Haematologica.

[40] Gertz MA, Comenzo R, Falk RH, Fermand JP, Hazenberg BP, Hawkins PN (2005). Definition of organ involvement and treatment response in immunoglobulin light chain amyloidosis (AL): a consensus opinion from the 10th international symposium on amyloid and amyloidosis, Tours, France, 18-22 April 2004. Am J Hematol.

[41] Palladini G, Merlini G (2019). When should treatment of AL amyloidosis start at relapse? Early, to prevent organ progression. Blood Adv.

